# rTMS Implementation in ELFT (East London Foundation Trust): A Prospective Clinical Study

**DOI:** 10.1192/bjo.2025.10212

**Published:** 2025-06-20

**Authors:** Areej Rasheed, Harith Ali, Asho Oommen, Daniel Allen, Geoff Lawrence-Smith

**Affiliations:** ELFT, London, United Kingdom

## Abstract

**Aims:** To evaluate the effectiveness and safety of repetitive transcranial magnetic stimulation (rTMS) in treating treatment-resistant depression (TRD), with a focus on changes in depression severity measured by the Montgomery–Åsberg Depression Rating Scale (MADRS) and the Hamilton Depression Rating Scale (HAMD).

**Methods:** A prospective clinical trial was conducted with 15 patients diagnosed with TRD, defined as having failed at least two adequate antidepressant trials. rTMS was administered using a left dorsolateral prefrontal cortex (DLPFC) protocol, with sessions delivered five times per week over six weeks for the majority of participants. Depression severity was assessed using MADRS and HAMD scores both before and after treatment. Adverse events were monitored throughout the study. Paired t-tests were used to analyse changes in MADRS and HAMD scores, with statistical significance set at p<0.05. Effect sizes were calculated using Cohen’s d.

**Results:** The average MADRS score decreased from 35.33 pre-intervention to 24.67 post-intervention, reflecting a mean reduction of 10.67 points and a large effect size (Cohen’s d=1.23). Similarly, HAMD scores decreased from 22.83 to 13.67, with a mean reduction of 9.17 points and a large effect size (Cohen’s d=0.98). While most patients demonstrated significant improvement, one patient experienced worsening symptoms. Adverse events were generally mild, with 7 patients reporting no side effects and 4 reporting mild pain at the stimulation site.

**Conclusion:** rTMS appears to be an effective and well-tolerated treatment option for reducing depressive symptoms in patients with TRD. The significant reductions in MADRS and HAMD scores, along with large effect sizes, support the potential of rTMS as a therapeutic intervention for this population. Further research with larger sample sizes, including the use of a control group, is needed to confirm these findings and explore the long-term efficacy of rTMS in managing TRD.

